# Petrox

**Published:** 1912-04-27

**Authors:** 


### THERAPEUTICS.

### Petrox.

PETROX or petroxolinum consists of an ammoniated derivative of oleic acid, and if the oleates are useful as external applications, the petrolates are still more so, owing to the fact that the ammoniacal soap of oleic acid readily emulsifies fats in water and leads to more rapid absorption through the skin than does oleic acid itself. The subject has been investigated by Messrs. G. M. Beringer, father and son, the results having been communicated to the Philadelphia College of Pharmacy, and published both in the *American Druggist* and in the *Pharmaceutical Journal* for May 20, 1911. Liquid petrox (petroxolinum liquidum) is a yellowish fluid, soluble in ether, chloroform, benzene, and acetone, and it produces an emulsion on agitation with twice its volume of water. The formulary for its preparation is as follows:—

| | |
|---|---|
| Liquid petrolatum | gm. 50.0 |
| Oleic acid | gm. 28.0 |
| Oil of lavender flowers | gm. 2.0 |
| Stronger ammonia water | gm. 5.0 |
| Alcohol | gm. 15.0 |

Mix the liquid petrolatum, oleic acid, and oil of lavender flowers in a flask, then add the alcohol, and finally the stronger ammonia water, and agitate thoroughly until clear, warming the mixture slightly on a water bath if necessary. Slight warming may be required in cold weather to promote the saponification.

This petrox may be used for the administration of all kinds of different substances externally, for instance, ichthyol, eucalyptol, iodoform, menthol, naphthol, phenol, sulphur, and so forth. One or two of the formularies given by Messrs. Beringer are as follows:—

PETROXOLINUM SULPHURIS. SULPHUR PETROX.

| | |
|---|---|
| Sublimed sulphur | gm. 3.0 |
| Linseed oil | gm. 37.0 |
| Oleic acid | gm. 30.0 |
| Liquid petroxolin, a sufficient quantity to make | gm. 100.0 |

Heat the sublimed sulphur and linseed oil in a flask, on a sand bath, until the sulphur is dissolved, then allow to cool and add the oleic acid and sufficient liquid petroxolin to make the product weigh 100 grammes, warming the mixture slightly if necessary to obtain a clear liquid.

PETROXOLINUM MENTHOLIS. MENTHOL PETROX.

| | |
|---|---|
| Menthol | gm. 5.0 |
| Liquid petroxolin | gm. 95.0 |

Dissolve the menthol in the liquid petroxolin by agitation.

PETROXOLINUM IODI. IODINE PETROX, 10 per cent.

| | |
|---|---|
| Iodine | gm. 10.0 |
| Oleic acid | gm. 40.0 |
| Alcohol | gm. 20.0 |
| Liquid petrolatum | gm. 23.0 |
| Oil of lavender flowers | gm. 2.0 |
| Stronger ammonia water | gm. 5.0 |

Reduce the iodine to a coarse powder by triturating in a glass mortar and transfer it to a suitable flask; add the alcohol and then the oleic acid, and agitate the contents of the flask until the iodine is dissolved; now add the oil of lavender flowers and the liquid petrolatum; mix the liquids, and finally add the stronger water of ammonia, shaking the mixture until a clear solution results.

